# Supplementary material for: Diverse Epidemiology of Leptospira Serovars Notified in New Zealand, 1999–2017
Source: Pathogens. 2020 Oct 14;9(10):841. doi: 10.3390/pathogens9100841 (PMC7602385; doi:10.3390/pathogens9100841)
Supplement: Supplementary file 1 [file pathogens-09-00841-s001.pdf]

pathogens

Supplementary Materials

Diverse epidemiology of *Leptospira* serovars notified in New Zealand, 1999-2017

Shahista Nisa [1], David A. Wilkinson [2], Olivia Angelin-Bonnet [3], Shevaun Paine [4], Karen Cullen [4], Jackie Wight [4], Michael G. Baker [5] and Jackie Benschop [1,6,*]

[1] Molecular Epidemiology and Public Health Laboratory, Hopkirk Research Institute, School of Veterinary Science, Massey University, Palmerston North, New Zealand; s.nisa@massey.ac.nz
[2] New Zealand Food Safety Science and Research Centre, Massey University, Palmerston North, New Zealand; david.wilkinson@univ-reunion.fr
[3] School of Fundamental Sciences, College of Sciences, Massey University, Palmerston North, New Zealand; Angelin-bonnet@massey.ac.nz
[4] Institute of Environmental Science and Research, Wellington, New Zealand; Shevaun.Paine@esr.cri.nz; karen.cullen@esr.cri.nz; Jackie.Wright@esr.cri.nz
[5] Department of Public Health, University of Otago, Wellington, New Zealand; michael.baker@otago.ac.nz
[6] Global Leptospirosis Environmental Action Network, World Health Organization, Geneva, Switzerland; J.Benschop@massey.ac.nz

* Correspondence: j.benschop@massey.ac.nz; Tel.: +64 6 359 9099 ext: 83994

Received: date; Accepted: date; Published: date

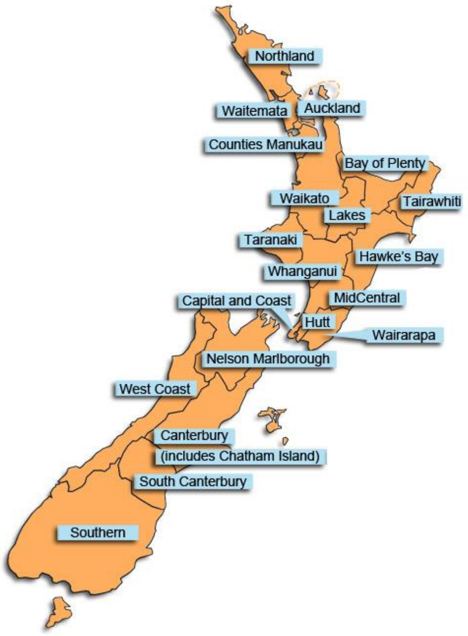

Figure S1: Location boundaries for the 20 District Health Board boundaries in New Zealand. Source: Ministry of Health, New Zealand.

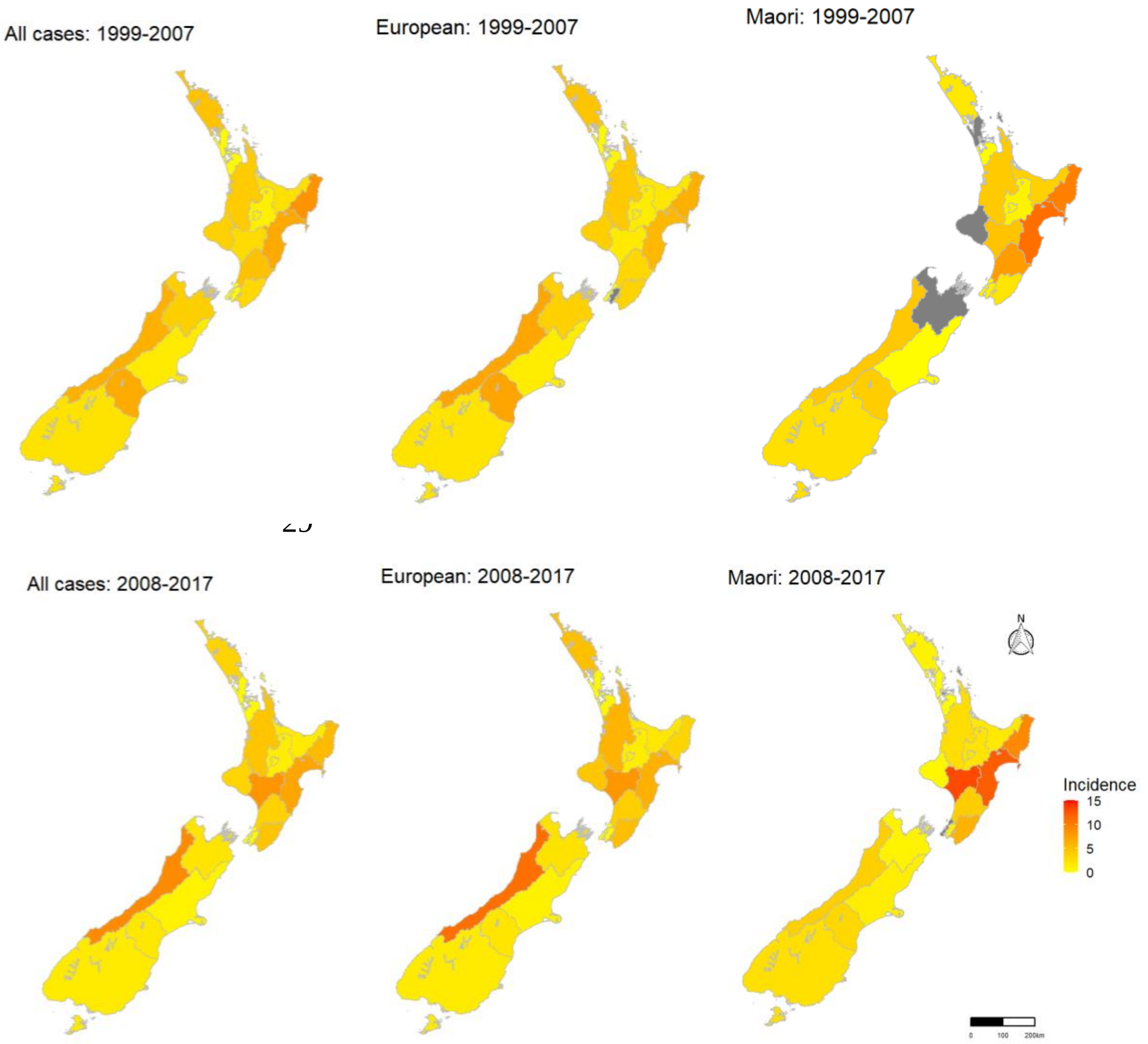

Figure S2: Choropleth maps of early and late study period. Average annual incidence per 100 000 of notified leptospirosis cases per District Health Boards in New Zealand for the early (1999-2007) and the late (2008-2017) study period for all cases, Europeans cases and Maori cases. Grey indicates DHB with no incidences.

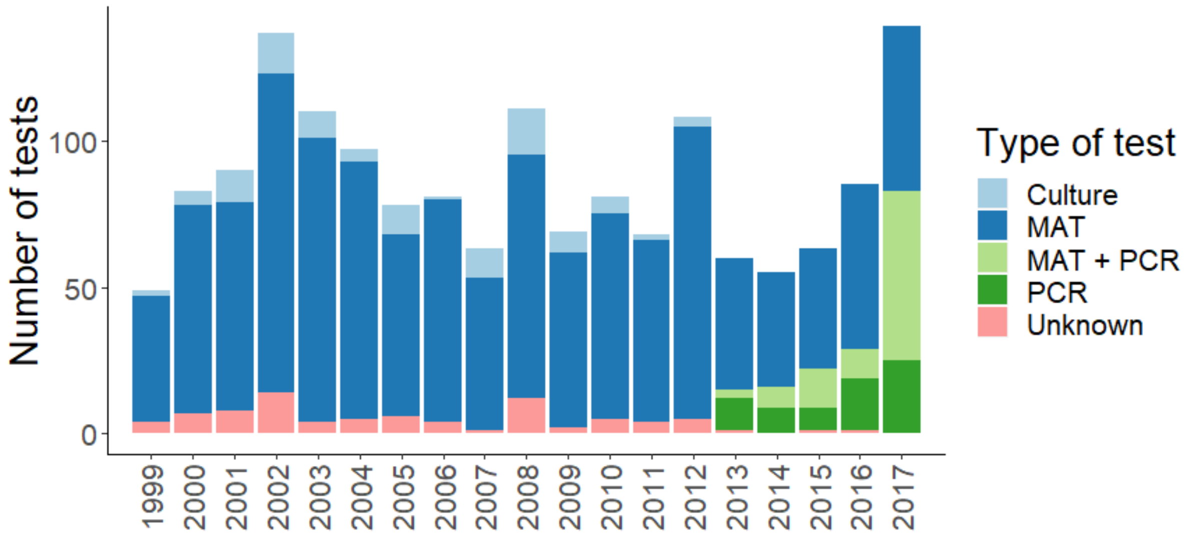

Figure S3: Type of laboratory tests used to diagnose notified confirmed and probable leptospirosis cases in New Zealand, 1999 to 2017.
